# Similar biomechanical properties of four tripled tendon graft models for ACL reconstruction

**DOI:** 10.1007/s00402-021-04030-8

**Published:** 2021-08-02

**Authors:** Davide Pavan, Federica Morello, Francesco Monachino, Giuseppe Rovere, Lawrence Camarda, Giuseppe Pitarresi

**Affiliations:** 1 Department of Orthopaedic Surgery, University of Palermo (DiChirOnS), Via del Vespro, 90100 Palermo, Italy; 2 Department of Orthopaedics and Traumatology, Fondazione Policlinico Universitario A. Gemelli, IRCCS-Università Cattolica del Sacro Cuore, Rome, Italy; 3 Department of Engineering, Università degli Studi di Palermo, Viale delle Scienze ed.8, 90128 Palermo, Italy

**Keywords:** Tripled graft, Triplicate, Graft diameter, ACL reconstruction, Graft, Knee biomechanics

## Abstract

**Purpose:**

The present study tested and compared the biomechanical properties of four different triplicate graft tendon techniques.

**Methods:**

32 tripled tendons from the common extensor muscle of bovine fingers were tested on a material testing machine, passing the end loop over a metal rod of a clevis connected to the load cell on the upper side, and fixing the lower end to a clamp. The samples were divided into four groups: (A) tripled with a free end sutured only to one of the two fixed bundles (B) tripled with a free end positioned between the two fixed strands and sutured to both (C) tripled with an S-shape and all the three strands sutured together at the upper and lower extremities of the graft (D) partially quadrupled with the free end sutured together with the other three bundles at the upper extremity. Each sample was pretensioned at 50 N for 10 min and then subjected to 1000 load control cycles between 50 and 250 N. Finally, each sample was subjected to a load to failure test.

Authors also present some preliminary results on the feasibility of a non-contact and full-field Thermoelastic Stress Analysis technique, based on Infrared Thermography, to evaluate the level of stress on the whole graft, and hence on each strand, during fatigue loading.

**Results:**

Eighty five percent of the samples failed at the level of the clamp. The cyclical elongation progressively decreased in all the samples and there was a simultaneous increase in stiffness. An increased stiffness was noted between Group 2 vs Group 3 and Group 2 vs Group 4 at the 500th and 1000th cycle. The failure loads were as follows: (a) 569.10 N, (b) 632.28 N, (c) 571.68 N, (d) 616.95 N. None of the parameters showed a statistically significant difference between the four groups.

**Conclusion:**

This study reported similar biomechanical behavior of four different models of tripled grafts suitable for ACL reconstruction. In addition, the biomechanics of overall tripled tendon grafts seems more affected by the viscoelastic property of the tendon itself rather than the preparation method.

## Introduction

Anterior Cruciate Ligament (ACL) Reconstruction is the gold standard for patients who develop knee instability after ACL rupture. Over the years, surgical reconstruction techniques have changed with technological progress and a deeper knowledge of graft biomechanics [1]. Furthermore, it has been observed that thicker grafts are associated with lower meniscal stress, decreased joint laxity, and less articular cartilage contact stress [2]. Recently, some authors have suggested that an increases of 0.5 mm up to a graft size of 10 mm are beneficial for the patient and a minimum diameter of 8 mm reduces the risk of graft failure [3, 4]. To obtain such sizes, allograft-based replacement surgeries avoid the problems of graft quantity and require reduced time for surgery and recovery since donor site morbidity is eliminated. On the other hand, it has an increased surgical cost and time to ligamentization [5, 6]. Donor medical history and sterilization processes of allografts can also affect the quality of the graft [7, 8]. Autograft in comparison incurs a slightly higher cost, is usually accessible, and is the gold standard graft especially in patients under 30 years of age [9]. A survey conducted among surgeons pointed out that the hamstring tendon was the first preferred choice of autograft, followed by the patellar tendon graft and allografts [10]. The main disadvantage in using the hamstring tendon autograft is the limited availability of tissue and the possible damage at the donor site [11]. In case of undersized tendons, one potential way to provide an adequate graft diameters is to triple hamstring grafts. Several authors [12–18] have presented mechanical assessment to clarify the properties of tripled grafts but the question remains open as to what is the best configuration that can maximize the cooperation effect between the strands.

The assumed of iso-stress condition among the strands has yet to be proved and little work is available about the influence of a number of external factors that can influence the ability of strands to sustain loads. These may include the ability of sutures to transfer and distribute loads uniformly among the strands, the initial pretension of each strand, and the role of the fixing and anchoring sites on the loading transfer to the strands [19].

The purpose of our study was to compare the biomechanical properties of four different methods of suture fixation to prepare tripled tendon grafts when a rigid suspensory fixation device is used. We hypothesized that the method of preparation of tripled tendon grafts does not affect the load to failure and displacement properties.

The work also presents some preliminary results of the use of Thermoelastic Stress Analysis [20] (TSA) to investigate the stress distribution among the strands of a tripled tendon, while being subjected to cyclic loading. TSA is based on the analysis of the frequency content of temperature maps, acquired over a certain time by means of an Infrared Thermal camera [21, 22]. The outcome of the signal processing is a map of the thermoelastic signal, i.e., a metric that is correlated to the local material volume change under elastic straining. Therefore, the thermoelastic map has the potential to reveal the zones of the tripled tendons that are more stressed. The full-field information and the non-contact type of measurement that characterize TSA, makes this technique beneficial and informative for the study of stress distribution in complex tendon assemblies, as proposed in this work.

## Materials and methods

The present study and the experimental protocol were approved and performed in accordance with the author’s Institution guidelines and regulation for the use of experimental animal tissue. Furthermore, the study was authorized by the local ethical committee (Department of Orthopaedic Surgery—DiChirOnS). Fresh-frozen bovine common digital extensor tendons were harvested from front legs of 8 mature bovine aging from 18 to 24 months [23]. Tendons were divided in half and selected to get 32 samples. Tendon grafts were then prepared and sized using a surgical blade to have an overall length of 27 cm and a diameter of 8 mm when the graft was tripled. In this phase, a diameter measurement tool was used (Smith and Nephew, Androver, USA). All tendon grafts were immediately wrapped in a physiological solution soaked gauze, stored at − 38 °C for a period of 5–7 days and then thawed at room temperature 12 h before use. As per standard intra-operative technique, each tendon end was separately whipstitched with no. 2 non-absorbable sutures (Ticron, Tyco, Waltham, MA) for a length of 30 mm [24]. Further, each tendon was folded and sutured creating a triple tendon graft. Graft preparation was performed by two Orthopaedic Surgeons, under a slight manual tensile load. This was performed to avoid permanent graft elongation that may affect the graft during loading, due to the slippage of the suture over the tendon tissue during cyclic loading.

Continuous saline graft irrigation using syringes was performed throughout the preparation and mechanical testing to prevent drying. For the tests, an electro-mechanic two-columns universal testing machine (Instron 3367), equipped with a 30 KN load cell (Instron Systems, Norwood, Massachusetts), was used. Since the experiments carried out in this work required load comprised between 50 and 700 N, the accuracy of the load cell was verified to be within ± 0.5% of the reading in the above range, which is considered sufficient for the evaluations made in this work.

**Fig. 1:**
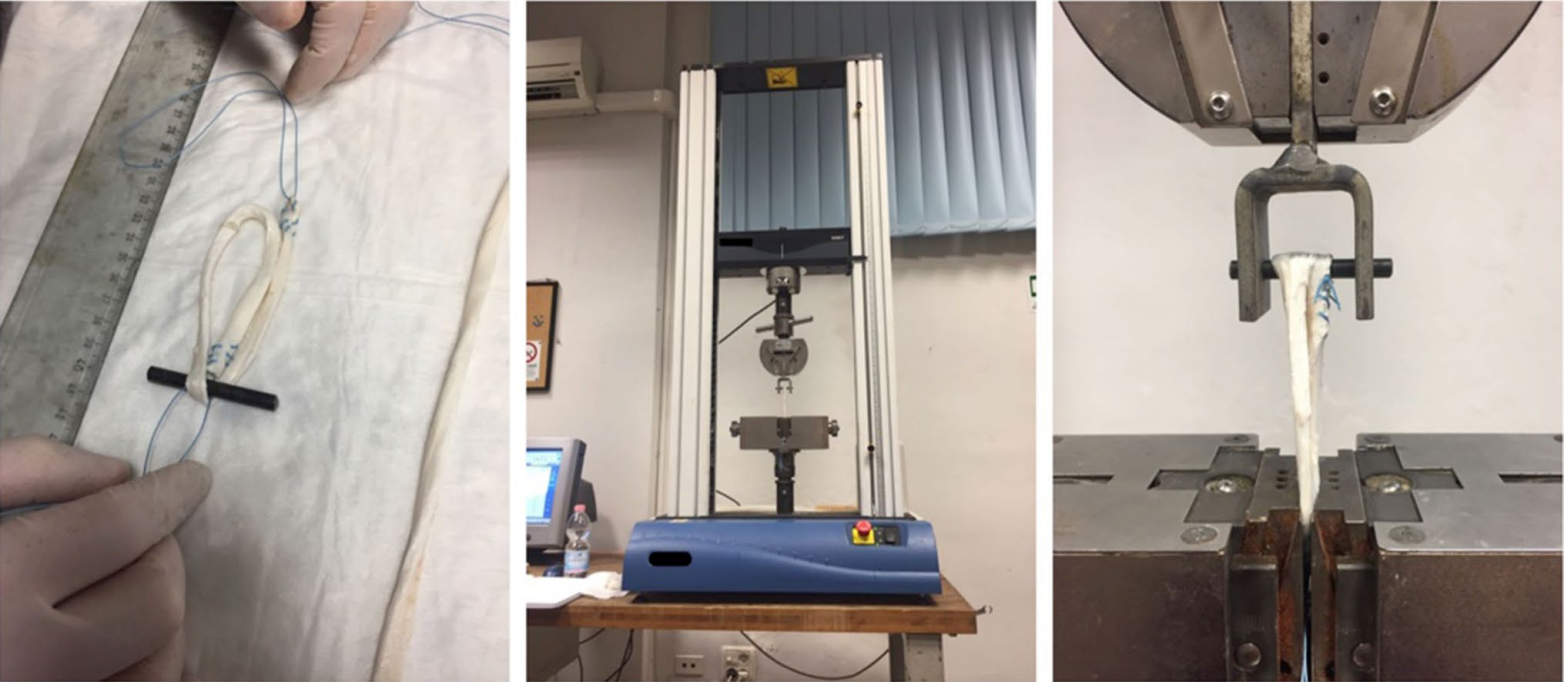
Experimental setup

**Fig. 2:**
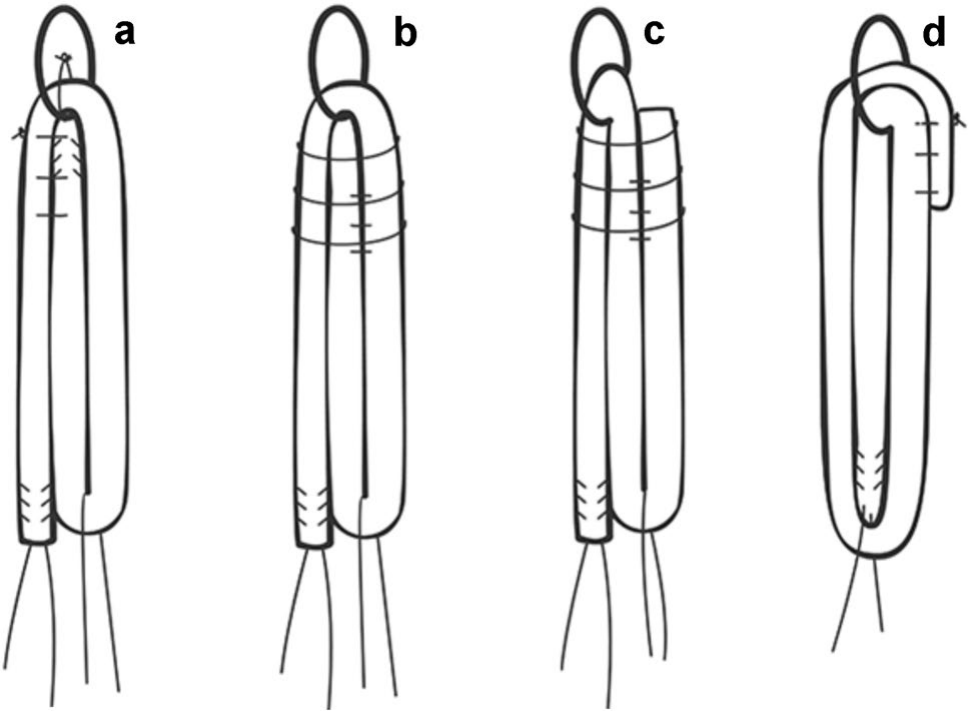
Illustration showing four different methods of folding and suturing the strands: **a** Group 1; **b** Group 2; **c** Group 3; **d** Group 4

The final tendon configuration (see Fig. 2) was obtained while connecting the tendon to the testing machine. In particular, regarding the connection of the upper part to the testing machine crosshead, each graft was passed over a cylindrical steel rod of 5 mm diameter, connected to a clevis preliminary clamped on an Instron mechanical wedge grip, which in turn was connected to the 30 KN load cell (Fig. 1b, c) [25, 26].

All couplings between the various parts where axially tightened by threaded flanges, so as to eliminate any axial clearance. The lower end of the samples was fixed by a Zwick Roell wedge-screw grip, clamping the samples for a length of 2 cm. The lower grip was firmly connected to the test machine rig, again eliminating any possible axial clearance. In this way, the crosshead displacement during the test could account only for the grafts stretching and/or slippage on the lower clamp.

Grafts were fixed with a distance of 70 mm from the clamp to the rod, to simulate the femoral tunnel length (40 mm) and intra-articular space of the ACL (30 mm) that could be obtained with more recent femoral fixation devices (Fig. 1).

Each tendon was tripled following four different methods of folding and suturing the strands, creating four different groups of eight grafts. These techniques of graft tripling were chosen after a literature review of all tripling graft techniques described so far.

### Group I

The tendon was looped around the rod with the free end positioned between the two fixed strands. The free end was sutured only to one of the two fixed bundles (Fig. 2a).

### Group II

The tendon was looped around the rod with the free end positioned between the two fixed strands and sutured to both (Fig. 2b).

### Group III

The tendon was looped around the rod with the free end positioned externally to the two fixed strands to form an inverted “S” configuration. All the three strands were sutured together at the upper and lower extremities of the graft (Fig. 2c).

### Group IV

Tendon was looped twice around the rod creating a partially quadruplicated graft with the free end sutured together with the other three bundles at the upper extremity (Fig. 2d).

Non-absorbable sutures (No. 2 Ticron, Tyco, Waltham, MA) were used for graft preparation. Each suture was passed under a slight tensile load of the graft, to avoid permanent graft elongation, due to the slippage of the wire over the tendon tissue during loads [27, 28]. The diameter of each graft construct was measured before tests using a diameter measurement tool was used (Smith and Nephew, Androver, USA) and a Vernier caliper with ± 0.05 mm accuracy.

### Biomechanical testing

**Fig. 3:**
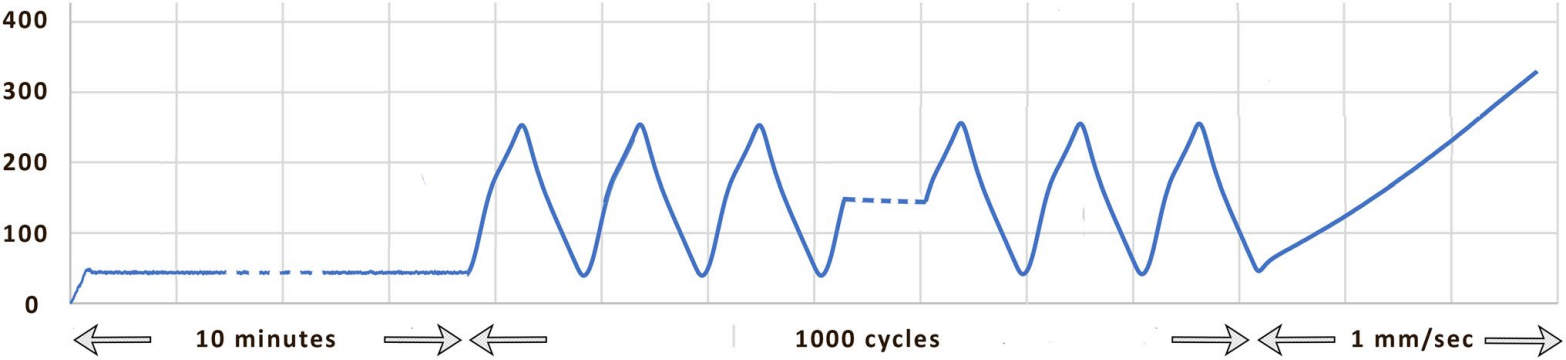
Scheme of the testing protocol, represented in terms of load versus time

A specific loading protocol was set up, by exploiting the Instron Blue Hill v 2.0 remote control software of the testing machine. The loading protocol set in this study comprised three successive stages (Fig. 3):

1. Static pre-conditioning by holding the tendon at a stable tensile load of 50 N for 10 min
2. Cyclic loading for 1000 cycles, between 50 and 250 N, with a triangle wave applied at 1 Hz;
3. Final monotonic tensile loading up to failure, performed in displacement control at a machine crosshead speed of 1 mm/s.

The pre-conditioning stage was performed to stabilize the graft’s mechanical properties [26, 29, 30]. Cyclic loading between 50 and 250 N with a frequency of 1 Hz was implemented to simulate the acting forces in the ACL flexion–extension during a walk [28, 29]. The number of 1000 cycles was chosen to simulate an intense postoperative rehabilitation protocol of the knee [25]. The final monotonic stage was intended to evaluate the residual static stiffness and strength bringing the graft to the breaking point.

For each specimen, load–displacement curves were recorded and analyzed to determine specific parameters. Specifically:

- The amplitude of graft elongation during a peak-to-peak fatigue cycle was determined, given by the difference in crosshead displacement between the load peak and the valley. For comparison purposes among the different lots of samples, the amplitude was measured at three stages of the cyclic loading window, and specifically: at the first applied fatigue cycle [L1], at the 500th cycle [L500] and at the last cycle [L1000].
- The graft slippage [L4] (the difference of graft accumulated elongation between the last and the first cycle, measured at the lowest point of the cyclic loading).
- The cyclic elongation [D0-500], defined as the difference in crosshead displacement between the condition at the end of the 50-N static pre-conditioning hold and the condition at the max applied load in the 500th fatigue cycle.
- The final elongation [D0-1000] (calculated at the 1000th cycle).
- The initial stiffness [K10], i.e. the slope of the secant line joining minimum and maximum points of the loading wave in the load–displacement curve, measured at the 10th cycle).
- Cyclic stiffness at 500th cycle [K500] (as described previously but at the 500th cycle).
- Pull-out stiffness [KL], i.e. the initial slope of the load–displacement curve at the final monotonic loading. The initial slope corresponds to the steepest straight-line tangent to the curve.
- Ultimate failure load [Fr], i.e. the peak force of the final load–elongation curve.

The mechanism of final static failure for each specimen was also observed and recorded.

### Thermoelastic stress analysis setup and testing

**Fig. 4:**
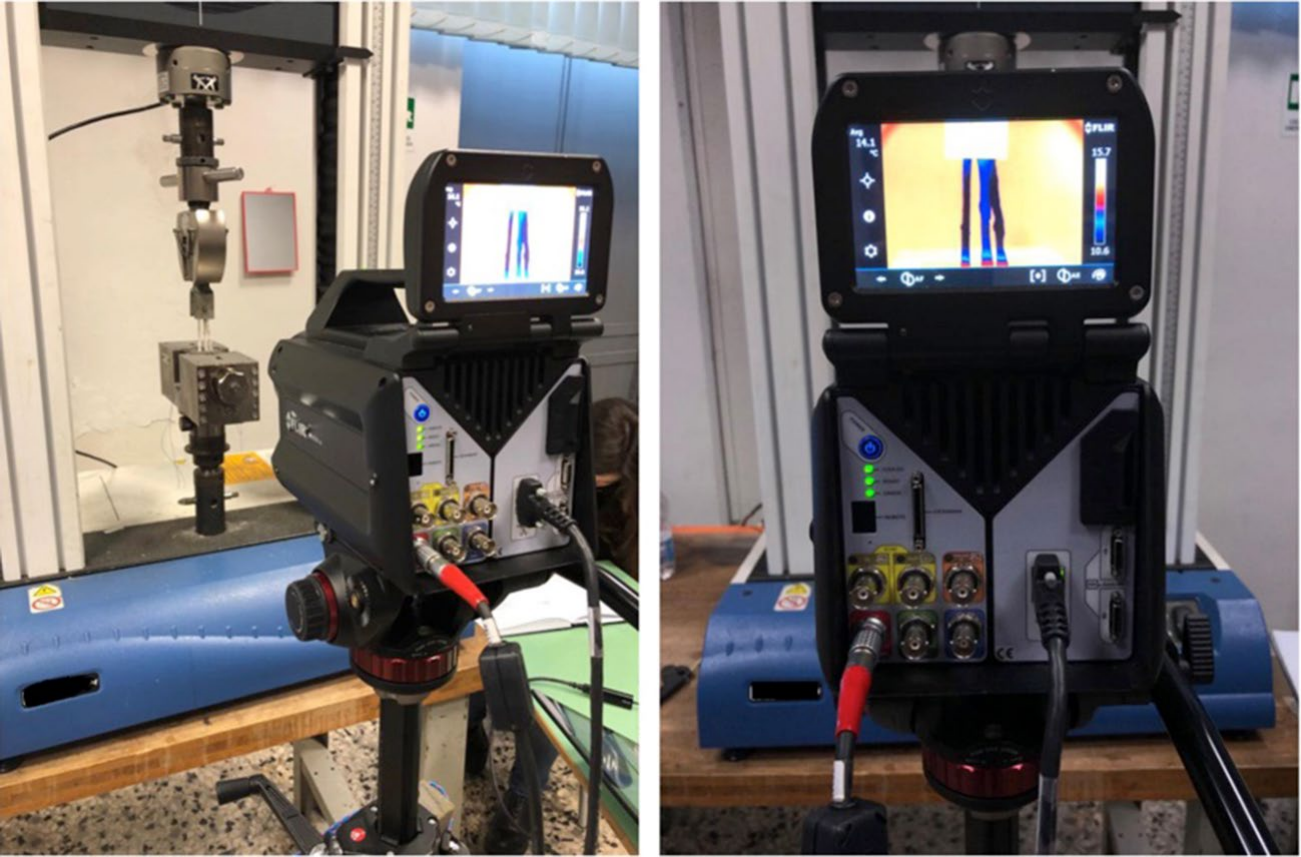
Experimental setup during Thermoelastic testing

Thermoelastic Stress Analysis (TSA) is a non-contact and full-field technique, based on the measurement of temperature by means of an Infrared Thermal camera, able to evaluate the level of elastic straining on solid matter when stressed under adiabatic conditions [20, 21]. In this study, the technique is proposed to evaluate the different levels of stress on tendon strands during fatigue loading (Fig. 4), where the cyclic application of the load is able to provide the required adiabatic conditions [21].

Thermoelastic Stress Analysis (TSA) exploits the thermoelastic effect observed in solid matter, according to which a solid structure, subject to elastic deformation, undergoes a volume change that produces a temperature change proportional to the stress metric that, for linear isotropic materials, coincides with the first stress invariant, i.e., the sum of the normal stresses. One peculiar feature of the thermoelastic effect is that it gives rise to a reversible temperature change. When a load is applied via a cyclic wave, this produces a temperature change that is modulated at the same frequency of the applied load. Therefore, the temperature changes filtered form the harmonic frequency content of temperature provides the thermoelastic signal, which carries the stress information needed.

The experimental implementation of TSA was performed in a couples of graft samples, using a high thermal resolution FLIR X6540sc IR camera, set with an integration time of 3000 ms, and acquiring thermograms during cyclic loading at a sampling framerate of 50 Hz and for a sampling time windows of 10 s. The thermoelastic acquisitions were randomly taken during the cycling stage of the tendons testing, and therefore the applied load consisted of a triangular wave at a frequency of 1 Hz. The temperature changes at the same frequency of the applied load (1 Hz) were measured from the acquired temperature by means a Discrete Fourier Transform-based signal post-processing. This allowed to filter all the temperature harmonics that had frequencies different from the load frequency. The central portions of all three strands of the graft should develop a uniform uniaxial stress change during load cycling, which is considered proportional to the measured thermoelastic signal. Therefore, TSA has the potential to reveal if and how the stress is redistributed among the three strands, by comparing the thermoelastic signal maps obtained. Furthermore, it is specified that no hydration was applied immediately before and during the few seconds of temperature measurement by the IR camera, since water behaves as a black body making the infrared signal coming from the tendons. Even so, a small degree of humidity, as left by initial hydration, at the start of the cyclic testing, was found to have no disturbing effects on the ability to perform TSA. Hydration may also cause the sample to undergo some slow temperature changes, such us cooling due to evaporation. These temperature changes, irreversible in nature, would not influence the thermoelastic signal since they are slow and not modulated at the load frequency, and therefore filtered out during the TSA signal processing.

### Statistical analysis

Data were analyzed using SPSS statistical software, version 18.0 (SPSS, Inc., Chicago, IL, USA). Means and standard deviations were calculated for all parameters of each group. Grafts tensile properties between the four graft groups were analyzed using the ANOVA method with a level of significance at *α* = 0.05. Further, each parameter (elongation, stiffness, slippage, load to failure, and failure displacement) was compared between groups using an independent *t* test, with a significant level placed at *p* < 0.05.

## Results

Each tripled graft was 9 cm long while the mean length of samples for each group from the rod to the clamp was 7.06 cm in Group I, 7 cm in Group II 6.87 cm in Group III, and 7 cm in Group IV. No differences in terms of graft diameter were observed among the 4 groups (> 0.05). All specimens completed phase 1 and phase 2 of the loading protocol (pre-conditioning and cyclical loading) and all graft failed during the final monotonic tensile load stage. In Group I, all samples failed at the level of the clamp; in Group II, rupture occurred at the level of the clamp in 75% of grafts and in the middle of the bundles in 25% of them.; in Group III and IV, 87.5% of the samples failed at the level of the clamp and in 12.5% failed in the middle of the grafts.

Concerning stiffness, a difference was observed at the 500th cycle between Group II vs Group III (263 vs 232 N; *p* = 0.013) and Group II vs Group IV (263 vs 225 N, *p* = 0.012). Further, a difference was observed at the 1000th cycle between Group II vs Group III (282 vs 253 N; *p* = 0.022) and Group II vs Group IV (282 vs 248 N, *p* = 0.029). No differences were noted between the four groups concerning all other parameters evaluated during cyclic loading (*p* > 0.05). Furthermore, no differences were noted between the four groups concerning pull-out stiffness and ultimate failure load (*p* > 0.05). The amplitude of graft elongation has gradually decreased simultaneously and consistently with the increase of the stiffness. All data and their related *p* values concerning statistical significance are represented in Table 1.

**Table 1:** Biomechanical properties of four different models of tripled grafts at cyclic loads and ultimate failure load

	Group 1 (a)	Group 2 (b)	Group 3 (c)	Group 4 (d)	*p *value
Amplitude 1 (mm)	1.10 ± 0.3	0.98 ± 0.1	1.06 ± 0.1	1.00 ± 0.1	0.42
Amplitude 500 (mm)	0.97 ± 0.3	0.81 ± 0.04	0.93 ± 0.1	0.90 ± 0.1	0.24
Amplitude 1000 (mm)	0.88 ± 0.2	0.74 ± 0.04	0.83 ± 0.1	0.81 ± 0.1	0.28
Stiffness 1 (N/mm)	192.20 ± 38.7	205.40 ± 17.8	194.36 ± 17.0	191.42 ± 21.4	0.67
Stiffness 500 (N/mm)	230.59 ± 51.7	263.08 ± 13.3 ^*,**^	232.79 ± 25.7 ^*^	225.01 ± 32.5 ^**^	0.13
Stiffness 1000 (N/mm)	248.64 ± 51.7	282.48 ± 14.2 ^*,**^	253.37 ± 27.5 ^*^	248.41 ± 34.7 ^**^	0.18
Elongation 1–500 (mm)	2.52 ± 0.7	2.74 ± 0.6	2.38 ± 0.5	3.18 ± 2.4	0.65
Elongation 1–1000 (mm)	2,81 ± 0.9	3.12 ± 0.9	2.76 ± 0.6	3.49 ± 2.8	0.77
Slippage (mm)	1.93 ± 0.9	2.38 ± 0.8	1.93 ± 0.6	2.68 ± 2.8	0.72
Failure displacement (mm)	8.43 ± 2.3	10.57 ± 1.9	9.26 ± 0.9	11.13 ± 3.9	0.14
Load to failure (N)	569.1 ± 107.8	632.3 ± 167.5	571.7 ± 101.5	615.9 ± 147.9	0.72

Data presented as mean ± standard deviation. No statistically significative differences were noted comparing groups (ANOVA). Concerning stiffness, *t* test showed a difference at the 500th cycle between Group 2 vs Group 3 (*p* = 0.013)* and Group 2 vs Group 4 (*p* = 0.012)**. A difference at the 1000th cycle between Group 2 vs Group 3 (*p* = 0.022)* and Group 2 vs Group 4 (*p* = 0.029)** was also observed

**Fig. 5:**
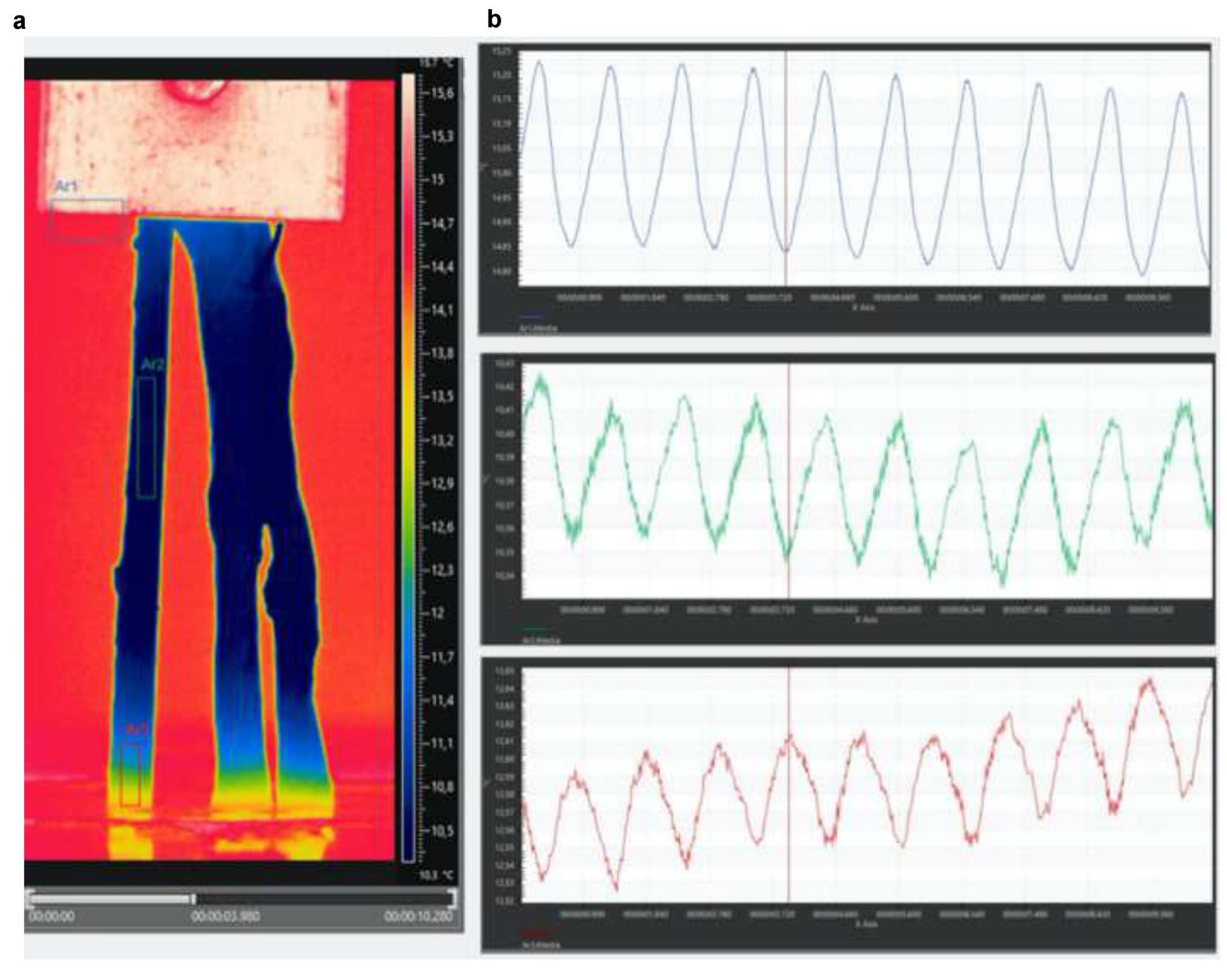
(Left) Example of a thermogram acquired by the IR camera during cyclic loading; (right) plots of average temperature versus time from three different zones highlighted in Fig. 5

Concerning the Thermoelastic Stress Analysis (TSA), the analyzed area comprised of the whole strand and part of the upper and lower connections to the testing machine. Figure 5a shows an example of a thermogram acquired during the time window acquisition. In this thermogram, three sub-areas are selected from which the average temperature was obtained and plotted versus time, as shown in Fig. 5b.

In particular, area Ar1 is taken across a horizontal border of the upper clevis, area Ar2 is taken on the central gage portion of one of the three strands, and area Ar3 is taken near the clamped lower end of the same strand. The area Ar1 is in part covered by the background and in part by the sidewall of the clevis. Since the clevis is warmer than the background, the signal acquired from Ar1 is modulated due to the stretching of the tendon. In particular, while the load is applied, a different amount of background and clevis wall is found to fill Ar1, and therefore, the average temperature versus time is an exact reproduction of the loading wave. When the wave has a peak temperature, this corresponds to the lower position of the clevis, i.e., lower load, while a trough temperature is achieved when the clevis is in its upper position, at higher load. The plots from areas Ar2 and Ar3 show that the temperature of the tendon is modulated according to the load, which is an unmistakable indication that a thermoelastic reversible temperature change component is present in the temperature signal. It is interesting to observe that the temperature change is in the same phase as the load in Ar2 and in the opposite phase (i.e., with a 180° shift) in Ar3.

**Fig. 6:**
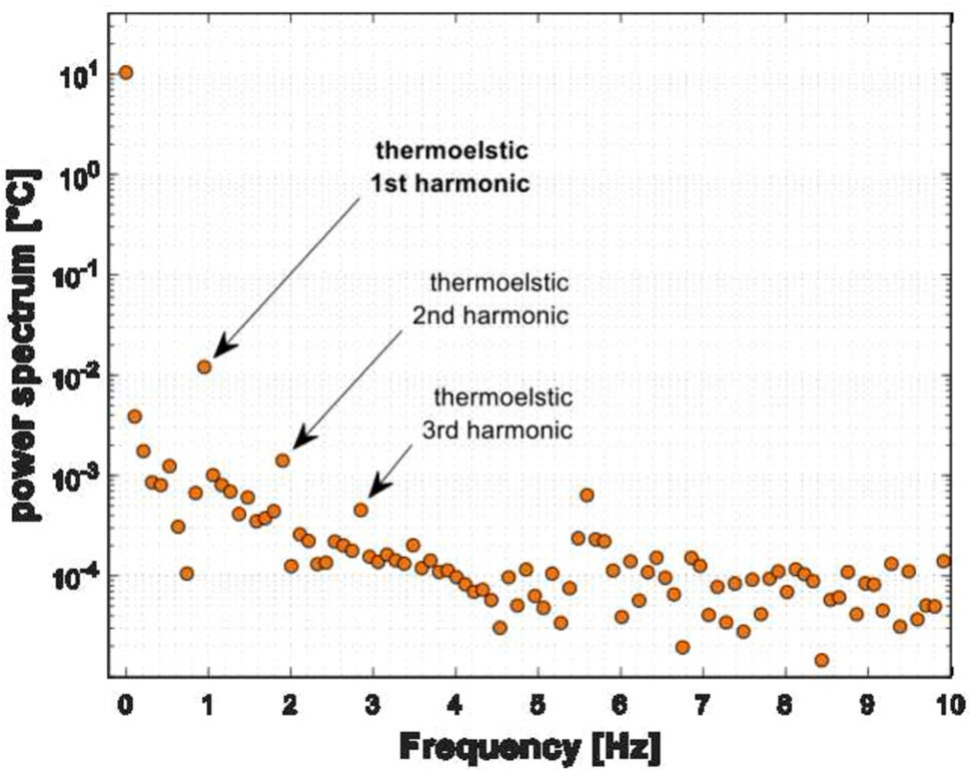
Power spectrum computed with the DFT of the signal from area Ar2

Figure 6 reports an example of a power spectrum, calculated with the Discrete Fourier Transform, of the average temperature signal from area Ar2.

The number of processed frames was modified to find the conditions for minimum spectral leakage at the expected frequency of the thermoelastic signal [31]. After this evaluation, the optimized frequency that carries the thermoelastic signal resulted to be about 0.95 Hz. This discrepancy with nominal 1 Hz load frequency is probably due to the electro-mechanic testing machine which is not very accurate in setting a user-specified load frequency (there is not a PID feedback control as in servo-hydraulic testing machines). In addition, the power spectrum confirmed the presence of a first, a second, and third harmonics (respectively, at which clearly emerge from the noise bed). This confirms that the triangular shape of the loading wave is reproduced in the thermal signal (first and third harmonic), and that a dissipative second harmonic term is also present as observed in more routinely in TSA applications on structural materials [31]. All these features ultimately suggest that the tendon is exhibiting a thermoelastic effect correlated behavior.

**Fig. 7:**
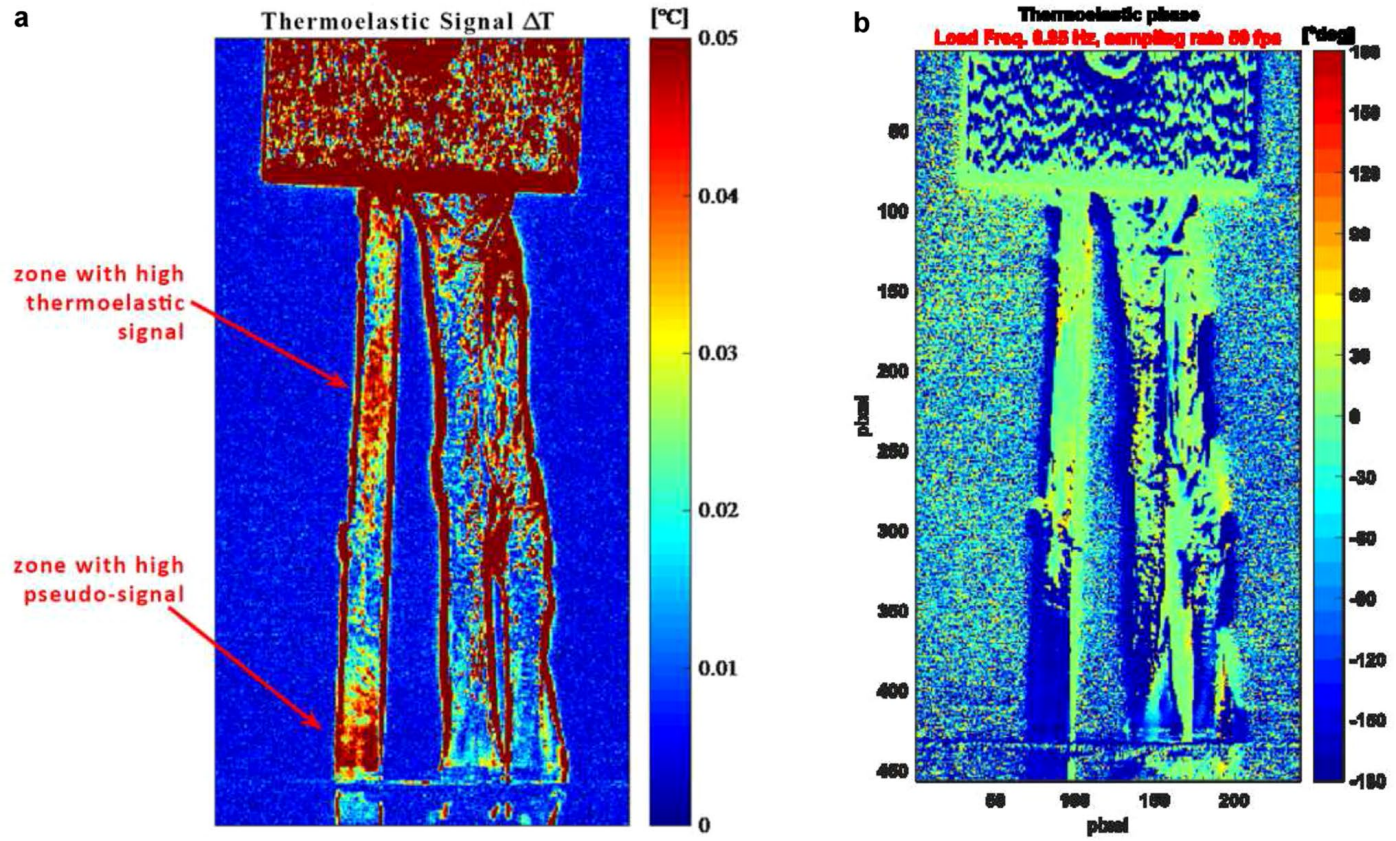
Full-field maps of thermoelastic signal amplitude (left) and phase (right)

Figures 7 provides the Thermoelastic signal amplitude maps and phase. It is immediately observed that a significantly higher thermoelastic signal was detected only on one single strand, and specifically the one where areas Ar2 and Ar3 were previously selected. The zones where the thermoelastic signal is higher are located at the level mid-gage length level and near the clamped zone.

## Discussion

The main finding of this study is that the biomechanical behavior of 4 different models of tripled grafts suitable for ACL reconstruction have been shown to be similar. Furthermore, we proposed an original implementation of an experimental stress analysis technique to evaluate the mechanical stress undergone by each strand of the grafts.

In our study, eight samples were tested for each group and this number is in line with current literature [16, 17, 30]. To standardize the graft preparation method, tendons were sized to have a diameter of 8 mm when the graft was folded in a tripled configuration. Even if the partially quadruplicated graft (Group 4) presented an increased graft diameter, no differences were noted between the four groups concerning all parameters evaluated during cyclic loading. This could suggest an unapparent biomechanical effect of a double looped tendon when a tripled graft is used. In this circumstance, increasing the femoral graft diameter seems to be not effective compared to tripled graft. Further, no differences were observed among all three techniques of tripling, suggesting that all techniques provide similar strand load distribution, leading to similar performances in terms of elongation during loading. An original aspect of our work is that the parameters collected not only concern the behavior at failure, but also monitor the biomechanical response evolution during cyclic loading. To the best of our knowledge, in the literature cyclic loads are applied to simulate a rehabilitation program, but only data related to ultimate failure load are generally available concerning tripled grafts [16, 17]. We believe that these measures, describing the dynamic behavior of grafts, allow a better characterization of the grafts and a more faithful comparison between the triplication methods.

Regarding the failure mechanism of the graft, in the study of Yoo et al. [17] and Fabbri et al. [32], the free tendon end was suggested as being the weakest point of graft for suspensory fixation because failure was due to tendon slippage across the suture. In our study, the weakest point in most of the samples, was located at the level of the clamp in most samples and this made us hypothesize that the method of fixation to the bone was decisive for the rupture of the sample. The most recurrent failure mode occurred at the clamp site, consisted mainly of gradual slippage, induced primarily by tearing the tendon near and inside the clamp. Therefore, most of the tested samples did not reach failure in the gage length, and the failure load cannot be associated to a material intrinsic tensile strength. Nonetheless, given the good repeatability of the failure mode observed, and the care used in trying to exactly replicate all testing conditions among samples, the values of load at failure are estimated to still meaningful and confirm the importance of the fixation site in determining the ultimate failure in an ACL reconstruction, regardless of the triplication technique.

In agreement with Fabbri et al. [32], stiffness maintained an overlapping value between the four techniques during the loading tests. In our work, also the amplitude of elongation showed a similar trend. The results from these parameters seem to be a proof that the biomechanics of overall tendon grafts is more affected by the viscoelastic property of the tendon itself rather than by the preparation method. An accepted assumption is that the effective overall strength/stiffness is improved by the joining together of strands, since these cooperate in resisting the loads, and lowering the stress on each single strand. However, it remains open the question about what is the best configuration that can maximize the above-mentioned cooperation effect, and thus exploit at best the presence of multiple strands [33]. The results of the present study could be helpful for surgeons in choosing graft configuration during ACL reconstructive surgery. Regardless of the triplication technique, the authors suggest paying attention during final graft fixation to obtain an equal tension in all three strands of the graft, improving strands cooperation, graft longevity, and effectiveness.

The implementation of TSA reported in this work is an attempt to evaluate the contribution of each tendon strand to resist to external loads. To our knowledge, no previous studies have tried to measure the thermoelastic effect induced temperature changes in tendons. For this reason, a Thermoelastic Stress Analysis technique was implemented, evaluating strands temperature change (Δ*T)* at the load frequency on a fatigue-loaded graft. The linear relationship describing the correlation between the temperature change at the load frequency and the stress change, for linear elastic and isotropic solid matter, can be written as follows:

$$\Delta T =  - {T_0}\kappa \Delta ({\sigma _{{\rm{xx}}}} + {\sigma _{{\rm{yy}}}})$$

where Δ*T* is the temperature change induced by the Thermoelastic Effect under adiabatic conditions and linear elastic material behavior. In Eq. (1) “To” is the initial body temperature, “k” a material constant and the stress term is the range of variation of the sum of in-plane normal stress components, i.e., the first stress invariant [19, 20].

In the present work, it was found that at the mid-length of the graft the left strand (sutured free-strand) has a significantly higher thermoelastic signal than the other two strands (Fig. 7a). This is believed to be due to the two strands on the right-hand side cooperating together in sustaining the portion of load distributed on the right-hand side. The total load is equally split between the left and right arms of the loop around the pin. Therefore, the left strand is over-stressed due to having to sustain alone the same portion of the load that is instead shared between two strands in the right-hand side of the graft. To our knowledge, this is the first study that has tried to evaluate tendon strands stress distribution using an optical, full-field and non-contact technique such as Thermoelastic Stress Analysis. This represents a preliminary report, and further work is under consideration to better understand and fully exploit the potentials of TSA to investigate tendon’s thermomechanical behavior and to better understand strands cooperation during loads. As reported in the results section, the authors believe that the presence of a thermoelastic effect response by the tendons is unmistakable as suggested by the reversible nature of the temperature fluctuation, and by its being in-phase or out-of-phase with the externally applied load, as suggested by the thermoelastic effect law (1). The thermoelastic map in Fig. 7 also shows that the left strand is the more stressed, with a higher thermoelastic signal showing up bot at the smaller mid-gage section and near the clamp. The presence of significant temperature gradients near the clamp zone in all three strands might suggest to believe that the high thermoelastic signal could instead be due to motion effects (thermoelastic pseudo-signal). However, if this was the case, the same high thermoelastic signal would be detected in all three strands while it really is much higher only on the left strand. Another interesting outcome is that the thermoelastic signals from the two areas Ar2 and Ar3 have a 180° shift with respect to the load. This indicates that the stress metric involved in making the thermoelastic signal has an opposite sign when considering the two sites. Further work is needed to allow for a more quantitative interpretation and correlation of the thermoelastic signal to a known stress metric. It is likely that the tendon material behavior is also highly orthotropic and that twist and transverse loads near the clamp zone might influence the thermoelastic behavior. Nonetheless, the qualitative data gained in this work from TSA, according to the authors, already represent a valuable and interesting result, proposing this technique as a way to investigate stress paths in a full-field, non-contact, and easy to implement manner.

Even if we tried to reproduce a scenario as similar as possible to the human one, this is only a laboratory biomechanical study and the comparison to the clinical practice could not be so immediate. First of all, we used bovine tendons for the mechanical simulations, however, some studies have shown a behavior similar to human tissue [23]. We used a clamp at the base of the grafts to simulate the tibial side, whereas during in vivo ACL reconstruction tibial fixation is commonly performed with interferential screws or cortical fixation devices. We used a rigid fixation because we aimed to evaluate the biomechanical properties of the tendon tissue itself even if we are conscious of the limitations of such choice. Furthermore, the knee joint moves in multiplanar directions during its movements and the tensile loads on the ACL grafts change continuously. In our study, the tensile tests were unidirectional and we did not reproduce such variability.

## Conclusion

This study reported similar biomechanical behavior of four different models of tripled grafts suitable for ACL reconstruction. The biomechanics of overall tripled tendon grafts seems more affected by the viscoelastic property of the tendon itself rather than the preparation method. Further, Thermoelastic Stress Analysis could represent an alternative technique to evaluate the mechanical stress experienced by each strand of the grafts during loads.
